# Evaluation of Antibacterial Activity of Essential Oils and Their Combination against Multidrug-Resistant Bacteria Isolated from Skin Ulcer

**DOI:** 10.1155/2021/6680668

**Published:** 2021-03-26

**Authors:** Eshetu Gadisa, Hydar Usman

**Affiliations:** ^1^Department of Medical Laboratory Science, MMHSC, KMU, Addis Ababa, Ethiopia; ^2^Department of Pharmacy, Health Science College, Madda Walabu University, Bale Robe, Ethiopia

## Abstract

**Background:**

Emerging of multidrug-resistant bacteria can compromise the effectiveness of antibiotics used to treat skin infections. Those bacteria imposed public health problems and questioning medical care in the 21^st^ century. In this circumstance, essential oils of medicinal plants origin are supreme sources of structural and functionally divergent compounds, which inhibited the growth of common wound colonizing MRSA and ESBL producing *P. aeruginosa.* The aim of this study was to evaluate the combined antibacterial activity of essential oils extracted from *Rumex abyssinicus, Cucumis pustulatus,* and *Discopodium penninervium* against multidrug-resistant (MDR) isolates of skin ulcers.

**Methods:**

Essential oils (EOs) were extracted from aerial parts of *R. abyssinicus, C. pustulatus*, and *D. penninervium* with steam distillation. A mixture of each oil (1 : 1) was adsorbed to a disc and placed on Mueller Hinton Agar. Then, minimum zone of inhibition and bactericidal concentration of EOs was measured after incubeted for 18–24 hours at 37 °C. Their combined antibacterial effect was determined by the fractional inhibitory concentration index.

**Results:**

The antibacterial activity of mixed oil varied in their doses and bacteria species, of which a mixture of essential oil of *R. abyssinicus* and *D. penninervium* had inhibition zone (32 mm); its MIC and MBC values range from 1-2 *μ*l/ml against MRSA. It had an inhibition zone (36 mm), MIC value 4 *μ*l/ml, and MBC (8 *μ*l/ml) against ESBL producing *P. aeruginosa*, whereas combined effects of *R. abyssinicus* and *C. pustulatus* had MIC values ranging from 2–8 *μ*l/ml for *E. coli* and *K. pneumoniae* and 2 *μ*l/ml for MRSA. There was a strong synergistic effect between *R. abyssinicus* and *D. penninervium* and promising antibacterial effect more specifically on MRSA and *P. aeruginosa. Conclusion*. This in vitro study of the combined effect of EOs has significant antibacterial activity on wound colonizing bacteria and reduces delaying wound healing as that of modern drugs tested in parallel. Hence, further structural elucidation of active compounds helps us to properly design or synthesis of topical antibiotics for wound care.

## 1. Background

Modern humans have universally used medicinal plants for healing properly many ailments [1, 2]. Evolving of the powerful analytical tools based upon proteomics, metabolomics, and genomics can aid to discover novel compounds from medicinal plants. The compounds of plant origin have divergent chemical structures and functionally relevant precursor molecules to discover antibiotics [2, 3]. Besides this, widely ethnobotanical and ethnopharmacological studies of essential oils from plants contributed to finding various compounds, of which leptospermone, tricyclene, flavesone, myrcene, carvacrol, p-cymene, eugenol, *γ*-terpinene, phenylpropanoids, *β*-selinene, and calamenene are some important compounds inhibiting the growth and biofilm formation of pathogen bacteria and used as immunomodulatory compounds [4, 5]. For instance, carvacrol caused collapse of the proton-motive force and depletion of the ATP pool, with consequent cell death [6]. Therefore, essential oils are applicable as precursors in the pharmaceutical industries for development of antibiotics [7, 8].

Nowadays, there is an increasing attention in exploring potential therapeutic bioactive to treat serious ailments caused by multidrug-resistant bacteria such as chronic wound [3, 4, 8, 9]. WHO reported that there were more than 8.2 million people infected with wounds with or without infection. This caused $28.1–96.8 billion lost to treat acute and chronic wounds. In this regard, the United States lost $25 billion per year for healthcare expenditure for nonhealing ulcer [10]. Likewise, a study conducted in Europe showed that there were 1·5–2 million people agonized from wounds. Unless and otherwise invented for new novel treatment, it imposed serious economic impact and costs $15–22 billion per year in coming five years. It has become major public health problem that caused psycho-social consequence on infected patients [9, 10]. Emerging of MRSA, VRE, and ESBL producing *P. aeruginosa* that identified as common wound colonized MDR bacteria has been worsen medical-surgical care and other health services [9–11]. In such case, essential oils of medicinal plants origin are suitable candidates to develop topical ointments for wound care and beyond [8, 12].

Essential oils have many compounds that can aid wound healing. In this regard, essential oil able to immunomodulating potential to typically activate both humoral and cell-mediated immune response. Other compounds such as carvacrol have bactericidal property through inhibition of protein and nucleic acid synthesis of MRSA and ESBL producing Enterobacteriaceae [5, 12, 13]. Some of them prevent biofilm formation and multiplication, inhibitory effect on inflammatory edema formation, and leucocyte chemotaxis around the wound. Therefore, essential oils are improving the quality wound care, decrease morbidity, and mortality and overcoming nonhealing trajectory and low therapeutic response of chronic wound infection [8, 12, 13].

Many studies showed that combined effect of essential oils had powerful antibacterial activity on wound colonizing pathogen multidrug-resistant bacteria [3, 12, 13]. The EOs of *Cinnamomum verum* and piperacillin mixture had synergistic activity against beta-lactamase TEM-1* E. coli*. This combination made wrecked the outer membrane or QS inhibition in bacteria cells [14]. Interestingly, a study conducted on Qingre Baidu mixture could inhibit the biofilm formation of *S. aureus* and *P. aeruginosa.* It reduces AI*-2* level and upregulating expression of HIF*-*1*α,* HIF-2*α,* and HIF-3*α,* which increased the levels of VEGF, thereby promoting angiogenesis and wound healing in chronic and refractory wounds [15]. Another study showed that EOs of *Pilgerodendron uviferum* and *Melaleuca alternifolia* can inhibit efflux pump mechanism of the *S. aureus* NorA. These essential oils can inhibit efflux pump and characterize a potentially safe and affordable ingredients to develop skin friendly ointment to wound colonizing multidrug-resistant bacteria [16–20].

Currently, using combined conventional antibiotics and essential oils or essential oils themselves as wound care ointments are promising strategies to overcome multidrug-resistant bacteria. MRSA and ESBL producing *E. coli* have resisted for amoxicillin, tetracycline, piperacillin, ofloxacin, and oxacillin [21–23]. Many studies showed that combined effect of *Leptospermum scoparium* and Tri-EDTA; *Mentha piperita and Micromelum integerrimum* and*;* cinnamon bark oil and cinnamaldehyde had synergistic antibacterial activity on MSSA*, E. coli,* MRSA, ESBL, and *P. aeruginosa* [24, 25]. From these viewpoints, we purposed to evaluate combined antibacterial activity of EOs extracted from *R. abyssinicus, C. pustulatus,* and *D. penninervium* against bacteria isolated from wounds. Essential oils had immunomodulatory compounds that serve as a baseline data to formulate and designing novel compounds and find out the scientific rationale for the combined effects of untapped medicinal plants used by different societies.

## 2. Materials and Methods

### 2.1. Study Area and Design

#### 2.1.1. Study Area

This study was conducted in the Seweyna woreda, Bale Zone. This woreda is located 437 km away from Robe (zonal town) in the northern east direction and 750 km from the capital city, Addis Ababa. An elevation extends from 400–1850 m above sea level and is located at coordinates latitude 7°19′60.00″ N and longitude 41°19′60.00″ E. Major rivers include the Mekenisa, Dare, Manduba, and Kurkura. According to the national land survey, Seweyna is covered by abundant pasture (46.3%), arable or cultivable (24.4%) and forest or heavy vegetation (24.1%). This district is the remotest area with no infrastructure (transport, hospital, and power supplies). There has been one health center and residents depend on traditional knowledge of TMP to treat ailments such as skin diseases, diabetes, STI cancer, hypertension, and impotence.

#### 2.1.2. Study Design and Period

An in vitro experimental study was carried out to evaluate the antibacterial effect of combined essential oils, *R. abyssinicus, C. pustulatus,* and *D. penninervium,* against MDR and their reference strains at KMU, Core Laboratory, from January to April, 2020.

### 2.2. Medicinal Plants Selection Criteria

Nowadays, pastoralist communities are deprived of modern medical care and depend on the traditional medicinal plants to treat many human and animals' ailments such as skin diseases, diabetes, hepatitis, and cancer. These medicinal plants were selected based on traditional knowledge of healers to treat bacterial infections such as eczema, gonorrhea, syphilis, pneumonia, scabies, and other skin infections commonly with *R. abyssinicus, C. pustulatus,* and *D. penninervium.*

### 2.3. Plant Collection and Extraction

Plant samples of *R. abyssinicus, C. pustulatus,* and *D. penninervium* were collected from sideways of Mekenisa and Kurkura rivers of Seweyna woreda. Each plant was euthanatized and deposited at the National Herbarium with *Rumex abyssinicus* voucher number (E-55/07), *Cucumis pustulatus* (E-45/07), and *Discopodium penninervium* (E-18/07) in the Department of Biology, Faculty of Natural and Computational Science, AAU. Well-grown aerial part of each plant was collected and extracted its essential oils with steam distillation using AMIO-37/04 model for 3–5  hours and stored in brown colored bottle vials at 4 °C as described by Eshetu [3].

### 2.4. Culture Media and Multidrug Bacteria

#### 2.4.1. Culture Media

Nutrient agar, TSY broth, MacConkey, MHA, MHB, BA, mannitol salt agar, chocolate agar, and other reagents were used to grow bacteria collected from wounds.

#### 2.4.2. Test Organisms

The reference bacterial species of *E. coli* (ATCC25922), *K. pneumoniae* (ATCC700603), *E. faecalis* (ATCC 29212) and *S. aureus* (ATCC 25923), *P. aeruginosa* (ATCC 27853), and their MDR strains were isolated from wounds of out-patients attending Menelik Hospital, Addis Ababa. Ciprofloxacin (5 *μ*g), gentamycin (10 *μ*g), cephalotaxine (30 *μ*g), cefotaxime (5 *μ*g), ceftazidime (10 *μ*g), cefoxitin (30 *μ*g), ceftriaxone (30 *μ*g), amikacin (30 *μ*g), cefuroxime (5 *μ*g), ceftriaxone (30 *μ*g), cloxacillin (5 *μ*g), and augmentin (30 *μ*g) were antibiotics as described in CLSI guideline [26].

### 2.5. Screening for Multidrug-Resistant Bacteria

Multidrug-resistant bacteria were isolated from wounds. All bacterial cultures were first grown on 5% BA plates at 37°C for 18–24 hrs before inoculation onto the MHA. Few colonies (3–5) of similar morphology of the respective bacteria were transferred with a sterile inoculating loop to a liquid medium until adequate growth of turbidity with McFarland in 0.5. Then, the bacterial suspension was streaked on MHA plates using a sterile swab in such a way as to ensure thorough coverage of the plates and a uniform thick lawn of growth following incubation. The susceptibilities of clinical isolates were tested by using the MHA containing a range of antimicrobial agents. Dilutions of overnight broth cultures were inoculated onto antibiotic-containing plates to yield final inoculums of approximately 10^6^ CFU per spot for *S. aureus, E. faecalis, K. pneumoniae, E. coli,* and *P. aeruginosa* according to disk diffusion methods as recommended by CLSI guidelines [3, 26].

#### 2.5.1. Screening for Gram-Negative Bacteria

Selected multidrug-resistant Gram-negative bacteria such as ESBL producing *E. coli* and *P. aeruginosa* were detected by double disk synergy test (DDST) as described by Jarlier [27]. Mueller Hinton Agar was inoculated with standardized inoculum using sterile cotton swab. Augmentin (20 *μ*g amoxicillin and 10 *μ*g of clavulanic acid, AMC) disk was placed in the center of the plate and test disks of cephalosporins (ceftazidime 30 *μ*g, ceftriaxone 30 *μ*g, and cefotaxime 30 *μ*g) and aztreonam 30 *μ*g disks were placed at 20 mm distance (center to center) from the amoxicillin-clavulanic acid disk before incubation. The plate was incubated overnight at 35°C. Enhancement of the zone of inhibition of any one of the four drug disks toward amoxicillin-clavulanic acid suggested the presence of extended-spectrum beta-lactamases. *K. pneumoniae* was screened for its resistance to more than two different classes of antibiotics following disk diffusion method as CLSI guidelines [3, 26, 28].

#### 2.5.2. Screening for Drug Resistant Gram-Positive Bacteria

In this study, cefoxitin was used as the marker of mecA/mecC mediated by methicillin resistant *S. aureus* and vancomycin (VRE) drug of choice for disk diffusion method as recommended by CLSI guidelines. On the other hand, the concentration values (MIC and MBC) and fractional inhibitory concentration index were determined by MHB broth microdilution method as described by Eshetu [3, 26, 29, 30].

### 2.6. Statistical Analysis

Statistical data were reading values of inhibition zones and concentration values (MIC and MBC) analyzed using SPSS, version 21. Each experiment value is expressed as mean ± S.D. Statistical significance was determined by student t-test.

Significance was determined by student's *t*-test. Values with *p* < 0.05 were considered significant.

## 3. Results

### 3.1. Antibacterial Effect of Modern Antibiotics

Majority of bacteria isolated from wounds were resistant to two or more antibiotics. In this regard, methicillin resistant *S. aureus* (MRSA) and ESBL producing *P. aeruginosa* were major wound colonizing bacteria. From Gram-positive bacteria, MRSA and VRE were resisted for amikacin, cefoxitin, amoxicine, ampicillin, and cefotaxime (Table 1). On the other hand, Gram-negative bacteria ESBL producing *E. coli* and *P. aeruginosa* were isolated from surgical wounds. Those Gram-negative bacteria have been undermining in the case of wound care, whereas these bacteria strains were unpredictably resisted for classical modern third-generation cephalosporins and penicillin classes (Table 1). In such cases, gene or gene products of those bacteria transfer intra- or interbacteria species compromised treatment options and worsen the future of medical care.

### 3.2. Antibacterial Effect of Essential Oils

This study revealed that testing essential oils had broad bactericidal activities. They inhibited growth of *E. faecalis, E. coli, P. aeruginosa, K. pneumoniae,* and *S. aureus* with zones of IZ ranging from 12–26 mm (Table 2). Thus, oils demonstrated an antibacterial effect against wound colonization MRSA and ESBL producing Gram-negative bacteria. Their effectiveness varied with the concentration and type of bacterial species. For instance, *R. abyssinicus* inhibited the growth of MRSA (23 mm) and ESBL producing *E coli* (22 mm) and *P. aeruginosa* (20 mm) at its 20 *μ*l/disc, whereas *D. penninervium* inhibited the growth of MRSA (21 mm) and ESBL producing *E coli* (18 mm) and *P. aeruginosa* (22 mm) at its 20 *μ*l/disc. These essential oils had an inhibitory effect on both reference strains and MDR bacteria with IZ ranging from 9–26 mm in diameter and MIC and MBC values ranging from 2–32 *μ*l/ml. The EOs of *R. abyssinicus, C. pustulatus,* and *D. penninervium* showed IZ in diameter values 23 mm, 19 mm, and 21 mm on the MRSA, respectively. Vancomycin resistant *E. faecalis* were highly susceptible for tested EOs as compared to other species.

The essential oil of *R. abyssinicus* was powerful antibacterial active against Gram-positive and Gram-negative bacteria in general and VRE and *K*. *pneumoniae* in particular with 25 mm IZ at 20 *μ*l/disc. Likewise, *R. abyssinicus, C. pustulatus,* and *D. penninervium* showed IZ 20 mm, 19 mm, and 22 mm, respectively, on ESBL producing *P. aeruginosa* that were second common bacteria isolates from wounds. Those EOs had MIC and MBC values ranging from 4–32 *μ*l/ml. Overall, tested EOs can supersede wound colonization and biofilm forming bacteria (Table 3).

### 3.3. Combined Antibacterial Effect of Essential Oils

This study revealed that combination of essential oils 1 : 1 ratio was more strong antibacterial effect than individual essential oil alone and even equivalent to modern drug of choice. The combined EOs inhibited the growth of MRSA and ESBL producing *E coli* and *P. aeruginosa* and their reference strains at 20 *μ*l/disc (Table 3), of which the combined EO obtained from *R. abyssinicus* and *D. penninervium* (1 : 1 ratio) exhibited the strongest antibacterial activities. It had IZ in diameters 32 mm, 34 mm, and 19 mm on MRSA, VRE, and ESBL producing *P. aeruginosa* and *E. coli* at 20 *μ*l/disc, respectively. This mixture had the lowest MIC and MBC values as compared to the other mixture. The MIC value for VRE was 1.0 *μ*l/ml and MRSA (2.0 *μ*l/ml). The MBC values were ranging from 1.0–8.0 *μ*l/ml for ESBL producing *P. aeruginosa* (Table 4). This combined oil showed powerful bactericidal effect as compared to modern antibiotics against tested reference and drug-resistant bacteria species.

The combination of *C. pustulatus* and *R. abyssinicus* essential oils had 29 mm IZ in diameter against both MSSA and MRSA. It showed an effective antibacterial effect on *K. pneumoniae* and *E. coli.* It had MIC values ranging from 2.0–8.0 *μ*l/ml for tested Gram-negative bacteria and 2.0 *μ*l/ml against MRSA. It had MBC value 4.0 *μ*l/ml for *K. pneumonia* and MRSA and 16 *μ*l/ml against ESBL producing *P. aeruginosa*. Similarly, the combined effect of EOs obtained from *R. abyssinicus* and *D. penninervium* had 29 mm and 22 mm IZ in diameter at 20 *μ*l/disc against *K. pneumoniae* and *E. coli,* respectively (Table 3). It had also 32 mm IZ in diameter on MSSA and MRSA. Interestingly, combined effect of three EOs within 1 : 1:1 had 34 mm, 37 mm, and 26 mm on MRSA, VRE, and ESBL producing *P. aeruginosa,* respectively. This combination had MIC and MBC ranging from 0.25–4.0 *μ*l/ml for tested bacteria. Overall, the combination of each EO had more potent antibacterial effect on MRSA and *K. pneumoniae, P. aeruginosa,* and *E. coli* as compared to currently available and affordable antibiotics (Table 1).

The fractional inhibitory concentration index (FIC index) revealed that the combined essential oils from *R. abyssinicus* and *D. penninervium* in 1 : 1 ratio had synergistic effect on MRSA, VRE, and *K. pneumoniae.* It had also adaptive effect on ESBL producing *E. coli* and *P. aeruginosa.* On the other hand, mixture of *R. abyssinicus* and *C. pustulatus* had synergistic effect on VRE and additive on MRSA and *K. pneumoniae*. Conversely, it had antagonistic effect on ESBL producing *E. coli* and *P. aeruginosa*. The mixture of essential oils from *C. pustulatus* and *D. penninervium* showed additive effect on MRSA and *K. pneumoniae.* Its FIC index values were 075 and 0.625 for MRSA and *K. pneumoniae,* respectively. Overall, MRSA was most susceptible for combined essential oil. In contrary to this, *P. aeruginosa* resisted bacteria as compared to other bacteria for combined oil (Table 5).

## 4. Discussion

This study revealed that MRSA, VRE, and ESBL producing *E. coli* and *P. aeruginosa* were human pathogen MDR bacteria isolated from wound samples. Those strains were compromised for all or the majority of antibiotics used in low- and middle-income countries (Table 1). Similarly, many studies showed an alarming increase in multidrug-resistant bacteria on a global scale [3, 21–23]. Their resistance genes or gene products transfer into intra-and interspecies by transformation, transduction, and conjugation in very dynamic and unpredictable phenomena [16, 27]. On the other hand, almost all hospitals in developing countries have no drug susceptibility facilities to diagnose patients and administrated clinically. In this way, infected wounds with multidrug-resistant bacteria have limited treatment options and escalating mortality, morbidity rates, and treatment costs [13, 15]. These are ways that caused them to infect and spreading at the community level. As a result, they imposed a potentially large health and socioeconomic burden on societies and worries about the future provision of medical-surgical and other healthcare services [10]. In such cases, traditional healers use their ingenious knowledge such as medicinal plants to treat many ailments including chronic wound ulcers [8–10]. Since pastoralist communities are deprived of modern medical care and depend on the medicinal plants, they are resourceful for generating knowledge to tackle multidrug-resistant microbes in general and wound colonizing species in particular [3]. Those communities extracted essential oils from medicinal plants and used as a topical ointment for wound care in rural pastoralist communities [12, 19].

These study findings showed that almost all tested essential oils had promising antibacterial activity on the multidrug-resistant pathogenic bacteria colonizing and triggering wound infection, of which MRSA was more susceptible to tested essential oils in combination and/or alone. Likewise, VRE was also inhibited by most tested essential oils at 20 *μ*l/disc. These findings substantiate earlier studies that the therapeutic agents derived from essential oils can devastate the cell wall of bacteria and later kill them. Many compounds such as leptospermone, tricyclene, flavesone, myrcene, carvacrol, p-cymene, and eugenol are able to activate humoral and cellular immunity [14, 15]. Some of those compounds reduce proinflammatory cytokine and TNF resulting in improvement of wound healing [5, 8, 23, 29]. Another study demonstrated that carvacrol of essential oil has bactericidal property [6]. It inhibits protein and nucleic acid synthesis of Gram-positive bacteria, more specifically MRSA and VRE [7, 11]. Therefore, essential oils are candidate to treat chronic skin ulcer [9, 10, 12].

With other respects of Gram-negative bacteria, essential oils inhibited the growth of ESBL producing *P. aeruginosa and E. coli* and *K. pneumoniae* isolate of an infected wound. Bactericidal activity of essential oils was remarkable and pronounced inhibitory effect on references and drug-resistant bacteria. This insight into the application of essential oils on wound can improve healing. On the other hand, doses, types, and bacteria species determine the effectiveness of oils. These findings agreeing with essential oils can reduce biofilm formation and multiplication and inhibit inflammatory edema formation and leucocyte chemotaxis around the wound [16, 17, 19]. There were several determinants of acquired resistance to conventional drugs. In line with this, *P. aeruginosa* and *E. coli* developed resistance genes to many available and affordable antibiotics [20, 25]. These are associated with spontaneous mutations that interfere with drug-target binding and compromise prodrug activation or cause over expression of the target [24, 31]. Other studies showed that there were intrinsic genes, acquired antibiotic resistance (expressed new trait), and caused the genetic change. The genetic changes can confer resistance to antibiotics by altering the target site of the drug, enzymatical inactivation of the drug, and preventing the drug from accessing the target sites. Those factors compromised all or third-generation cephalosporins and penicillin classes [15]. Overall, those factors contributed to resisted amoxicillin, tetracycline, piperacillin, ofloxacin, and oxacillin [16, 18, 20].

This study revealed that combined effect of essential oils was more effective and efficient antibacterial effect than used alone and even equal to modern drug of choice. These synergistic combinations signify to exploited novel compounds from essential oils. This is also the enhanced bactericidal effect of the compounds. In such case, essential oils are suitable candidates to develop topical ointments for wound care and beyond [9]. Another study demonstrated that EOs caused sequential inhibition of biochemical pathway, inhibit protein synthesis, and disintegrated the outer cell membrane [18, 28]. Overall, essential oils have compounds that used to produce affordable and safe antibiotics and potentially activate the immune system for fast wound healing. The EOs have therapeutic compounds that exert beneficial pharmacological potential on wound care to overcome the problem from multidrug-resistant bacteria.

## 5. Conclusions

This study revealed that testing combined effect of essential oils had broad bactericidal activities, of which they demonstrated more strong antibacterial effect against common wound colonization of MRSA and ESBL producing *P. aeruginosa.* Moreover, majority of combined oil had synergistic and additive effect which provided information to search a novel compound in combination of essential oils or alone. Hence, identifying and structural elucidation of compounds with immunomodulatory and bactericidal effect from essential oils help pharmaceutical companies and researchers to develop less toxic, safe, and affordable drugs or modulators or precursors for the synthesis of new antimicrobial drugs and/or ointment to treat wounds.

## Figures and Tables

**Table 1:** Antibiotics resistant profile of multidrug-resistant bacteria isolated from wounds at KMU, 2020.

Modern drugs	Drug susceptibility test for multidrug bacteria strains isolated from wound samples														
	Ef1	Ef2	Ef3	A1	A2	A3	Ec1	Ec2	Ec3	Ar1	Ar2	Ar3	K1	K2	K3
Ciprofloxacin	R	R	I	R	I	R	R	R	R	R	S	I	R	I	R
Cefoxitin	R	I	R	R	R	R	N	N	N	N	N	N	N	N	N
Cefuroxime	S	R	I	N	N	N	R	R	R	R	R	R	I	R	I
Ceftriaxone	S	I	R	N	N	N	I	R	R	R	R	R	R	R	R
Ceftazidime	N	N	N	N	N	N	R	I	R	R	R	R	R	S	I
Chloramphenicol	R	R	R	S	R	I	R	R	S	R	R	R	S	R	R
Ampicillin	I	S	S	R	R	R	S	R	R	S	R	R	R	R	R
Augmentin	R	I	S	R	I	S	R	R	I	R	S	R	R	I	S
Amikacin	R	R	R	R	R	R	N	N	S	R	R	R	I	R	S
Erythromycin	R	I	R	R	R	I	R	I	R	R	R	R	S	S	R
Tetracycline	R	S	I	R	R	R	I	I	R	R	R	R	S	R	I
Penicillin	R	I	R	R	R	R	R	R	R	R	R	R	R	I	R
Vancomycin	R	R	R	I	R	S	N	N	N	N	N	N	N	N	N
Gentamycin	R	I	S	R	R	R	S	R	R	S	R	I	S	R	R

A = methicillin resistant *S. aureus;* Ar = ESBL producing *P. aeruginosa;* Ec = ESBL producing *E. coli;* N = not done; Ef = Vancomycin resistant *E. faecalis;* K = multidrug-resistant *K. pneumoniae;* I = intermediate (17–20 mm IZ); R = resistance (≤16 mm IZ); S = sensitive (≥21 mm IZ) according to CLSI guidelines [25].

**Table 2:** Inhibition zone (mm) of essential oils against MDR bacteria isolated from wounds at KMU, 2020.

Essential oils, 50 *μ*l/ml	*μ*l/disc	Inhibition zone in diameter (mm), pathogen bacteria isolated from wounds									
		Gram-positive bacteria				Gram-negative bacteria					
		*S. aureus*		*E. faecalis*		*E. coli*		*K. pneumoniae*		*P. aeruginosa*	
		ATCC	MRSA	ATCC	VRE	ATCC	ESBL	ATCC	MDR	ATCC	ESBL
*R. abyssinicus*	20	23 ± 0.8	23 ± 3.0^*∗*^	25 ± 1.3	25 ± 1.5^##^	21 ± 0.7	22 ± 0.2₫	25 ± 0.5	25 ± 0.9^$^	21 ± 0.8	20.9
	10	17 ± 0.4	18 ± .0.1	20 ± 0.9	19 ± 1.0	11 ± 0.3	12 ± 1.4	17 ± 1.7	17 ± 0.1	14 ± 1.2	14 ± 2.1
	5	10 ± 1.7	9 ± .0.9	14 ± 1.0	13 ± 1.5	NI	NI	11 ± 0.6	10 ± 1.1	8 ± 2.0	9 ± 0.1

*D. penninervium*	20	20 ± 2.0	21 ± 0.5^*∗*^	22 ± 0.1	22 ± 1.0^#^	19 ± 0.8	18 ± 0.1	26 ± 0.7	26 ± 0.4^$^	21 ± 0.9	22 ± 0.3^ ¥^
	10	13 ± 0.1	14 ± .0.3	17 ± 0.4	16 ± 0.7	14 ± 0.7	15 ± 1.0	18 ± 0.1	17 ± 0.5	17 ± 0.6	17 ± 0.7
	5	7 ± 0.7	8 ± .0.9	11 ± 0.1	9 ± 1.3	9 ± 1.6	8 ± 1.0	12 ± 1.9	9 ± 0.1	NI	NI

*C. pustulatus*	20	19 ± 0.1	19 ± 0.2	20 ± 0.1	21 ± 0.1^#^	12 ± 0.7	12 ± 1.1	22 ± 0.3	22 ± 0.4	19 ± 0.3	19 ± 0.4
	10	10 ± 0.5	9 ± 0.2	12 ± 0.6	14 ± 0.6	NI	NI	13 ± 0.4	13 ± 0.9	8 ± 0.4	11 ± 0.9
	5	NI	NI	9 ± 0.6	8 ± 0.3	NI	NI	7 ± 0.6	10 ± 0.6	NI	NI

Positive control		S	R	S	R	S	R	S	R	S	R
Negative control (5% DMSO)		NI	NI	NI	NI	NI	NI	NI	NI	NI	NI

Mean ± SD, C = ciprofloxacin; NI = no inhibition zone; NT = not tested; ATCC = susceptible >21 mm IZ, resistant <16 mm IZ, where *P* < 0.05 when compared to cefoxitin-treated MRSA, while ^#^*P* < 0.05 and ^##^*P* < 0.01 when compared to modern drug treated *E. faecalis* and ^$^*P* < 0.05 when compared to *K. pneumoniae*; ^ ¥^*P* < 0.05 when compared to modern drug treated *P. aeruginosa*, and ^₫^*P* < 0.05 when compared to modern drug treated *E. coli*.

**Table 3:** Inhibition zone (mm) of combined essential oils against MDR bacteria isolated from wounds at KMU, 2020.

Essential oils, 50 *μ*l/ml	*μ*l/disc	Inhibition zone in diameter (mm), pathogen bacteria isolated from wounds									
		Gram-positive bacteria				Gram-negative bacteria					
		*S. aureus*		*E. faecalis*		*E. coli*		*K. pneumoniae*		*P. aeruginosa*	
		ATCC	MRSA	ATCC	VRE	ATCC	ESBL	ATCC	MDR	ATCC	ESBL
*R. abyssinicus* and *C. pustulatus*	20	29 ± 2.1	29 ± 1.3^*∗*^	31 ± 1.3	31 ± 0.6^#^	23 ± 2.7	22 ± 1.7	33 ± 0.4	33 ± 0.1^$$^	26 ± 0.7	26 ± 0.9^ ¥^
	10	19 ± 0.5	16 ± .2.1	20 ± 0.7	21 ± 1.4	17 ± 0.4	18 ± 1.0	22 ± 2.0	23 ± 1.9	18 ± 1.1	18 ± 1.4
	5	13 ± 0.7	14 ± .0.5	10 ± 0.6	11 ± 0.6	10 ± 0.3	9 ± 1.4	15 ± 0.7	16 ± 2.4	11 ± 0.8	8 ± 0.4

*C. pustulatus* and *D. penninervium*	20	27 ± 1.0	25 ± 0.5^*∗*^	33 ± 0.1	32 ± 1.9^##^	27 ± 1.4	28 ± 0.1₫	32 ± 0.1	32 ± 0.7^$$^	28 ± 0.7	29 ± 0.6^ ¥^
	10	19 ± 0.9	17 ± .0.1	27 ± 1.5	27 ± 0.1	19 ± 0.4	19 ± 1.9	27 ± 1.3	26 ± 0.5	18 ± 2.4	17 ± 0.4
	5	12 ± 0.3	11 ± .0.8	17 ± 0.4	13 ± 0.8	9 ± 0.3	10 ± 0.7	13 ± 0.5	14 ± 1.3	12 ± 0.1	11 ± 1.5

*D. penninervium* and *R. abyssinicus*	20	32 ± 1.1	32 ± 1.2^*∗∗*^	34 ± 0.4	31 ± 2.1^##^	23 ± 1.3	22 ± 0.4	29 ± 0.3	29 ± 0.4^$^	21 ± 0.3	19 ± 0.4
	10	26 ± 2.5	26 ± 0.3	25 ± 0.6	24 ± 0.9	17 ± 1.7	16 ± 0.8	20 ± 0.6	20 ± 0.4	13 ± 0.4	14 ± 0.9
	5	13 ± 2.1	14 ± .0.8	16 ± 0.4	17 ± 0.7	9 ± 1.7	8 ± 0.5	15 ± 0.2	16 ± 0.7	8 ± 1.8	8 ± 1.3

Combination of three (1 : 1 : 1 ratio)	20	34 ± 0.3	33 ± .1.5^*∗∗*^	37 ± 0.2	37 ± 1.7^##^	22 ± 1.1	22 ± 1.6	35 ± 1.7	35 ± 0.2^$$^	26 ± 0.8	26 ± 0.4^ ¥^
	10	26 ± 0.1	26 ± .1.1	24 ± 0.8	25 ± 0.9	15 ± 1.2	16 ± 0.1	25 ± 2.0	25 ± 1.4	17 ± 0.5	17 ± 0.2
	5	17 ± 1.2	16 ± .0.9	18 ± 0.7	18 ± 0.8	9 ± 0.7	9 ± 0.4	16 ± 0.3	17 ± 0.9	12 ± 0.1	11 ± 1.9

Positive control		S	R	S	R	S	R	S	R	S	R
Negative control		NI	NI	NI	NI	NI	NI	NI	NI	NI	NI

Mean ± SD, whereas C = ciprofloxacin; NI = no inhibition zone; NT = not tested; ATCC = susceptible >21 mm IZ, resistant <16 mm IZ, where ^*∗*^*P* < 0.05 and ^*∗∗*^*P* < 0.01 when compared to cefoxitin treated MRSA, while ^#^*P* < 0.05 and ^##^*P* < 0.01when compared to modern drug treated *E. faecalis;*^$^*P* < 0.05 and ^$$^*P* < 0.01 when compared to *K. pneumoniae* and ^ ¥^*P* < 0.05 when compared to modern drug treated *P. aeruginosa,* and ^₫^*P* < 0.05 when compared to modern drug treated *E. coli*.

**Table 4:** MIC and MBC values of essential oils against human pathogenic bacteria in agar dilution method at KMU, 2020.

Essential oils extract	Concentratio*n* value	Concentration in *μ*l/ml on human pathogen bacteria isolated from wounds									
		Gram-positive bacteria				Gram-negative bacteria					
		*S. aureus*		*E. faecalis*		*E. coli*		*K. pneumoniae*		*P. aeruginosa*	
		ATCC	MRSA	ATCC	VRE	ATCC	ESBL	ATCC	MDR	ATCC	ESBL
*D. penninervium*	MIC	16.0	16.0	8.0	8.0	16.0	16.0	2.0	4.0	4.0	8.0
	MBC	16.0	32.0	8.0	16.0	32.0	32.0	2.0	4.0	2.0	8.0

*R. abyssinicus*	MIC	4.0	4.0	4.0	4.0	16.0	16.0	16.0	16.0	16.0	16.0
	MBC	4.0	8.0	8.0	8.0	16.0	16.0	16.0	16.0	16.0	32.0

*C. pustulatus*	MIC	8.0	8.0	16.0	16.0	32.0	32.0	4.0	4.0	16.0	16.0
	MBC	8.0	16.0	16.0	16.0	16.0	16.0	4.0	8.0	32.0	32.0

*D. penninervium* and *C. pustulatus*	MIC	4.0	4.0	1.0	2.0	16.0	16.0	1.0	1.0	16.0	16.0
	MBC	4.0	8.0	1.0	4.0	4.0	4.0	1.0	2.0	32.0	16.0

*C. pustulatus* and *R. abyssinicus*	MIC	2.0	2.0	4.0	4.0	8.0	8.0	2.0	2.0	8.0	8.0
	MBC	2.0	4.0	8.0	8.0	8.0	8.0	1.0	4.0	16.0	16.0

*D. penninervium* and *R. abyssinicus*	MIC	1.0	1.0	1.0	1.0	4.0	4.0	1.0	1.0	2.0	8.0
	MBC	2.0	2.0	1.0	1.0	2.0	4.0	1.0	1.0	4.0	4.0

Combination of three (1 : 1 : 1 ratio)	MIC	0.5	1.0	0.25	0.25	2.0	2.0	0.5	1.0	2.0	2.0
	MBC	1.0	1.0	0.25	0.25	1.0	2.0	0.25	0.5	2.0	4.0

Modern drug											

ATCC = reference strain for each species; MDR = multidrug-resistant strains; MBC = minimal bactericidal concentration; MIC = minimal inhibitory concentration; NT = not tested; *C* = 5 *μ*g ciprofloxacin +growth has been seen.

**Table 5:** The mean fractional inhibitory concentration index for MDR bacteria at KMU, 2020.

Mixture of oil in 1 : 1 ratio	Gram positive		Gram negative		
	MRSA	VRE	MDR *K. pneumoniae*	ESBL producing bacteria	
				*E. coli*	*P. aeruginosa*
*D. penninervium*	0.75^*∗*^	1.25^#^	0.625^*∗*^	0.75^*∗*^	1.0^*∗*^
*C. pustulatus*					

*C. pustulatus*	0.75^*∗*^	0.375^ ¥^	0.5^*∗*^	1.5^#^	3.0^#^
*R. abyssinicus*					

*D. penninervium*	0.3125^ ¥ ¥^	0.375^ ¥^	0.3125^ ¥^	0.5^*∗*^	0.5^*∗*^
*R. abyssinicus*					

The values represent mean fractional inhibitory concentration index (*n* = 6), where ^ ¥ ¥^*X* = synergy interaction, ^ ¥^*X* = partial synergy, ^*∗*^*X* = indifference interaction, and ^#^*X* = antagonistic interaction of essential oil.

## Data Availability

All data and materials of this work are available from the corresponding author on request.

## Abbreviations

ATC: American Type Culture Collection used as reference strains for respective bacteria
BA: Blood agar
CLSI: Clinical Laboratory Standard Institute
DDST: Double disk synergy test
EOs: Essential oils obtained from the respective medicinal plant by steam distillation
ESBL: Extended-spectrum beta-lactamase
FIC: Fractional inhibitory concentration index
MBC: Minimum bactericidal concentration
MDR: Multidrug resistant
MHA: Mueller Hinton Agar
MHB: Mueller Hinton Broth
MRSA: Methicillin resistant *Staphylococcus aureus*
MBC: Minimal bactericidal concentration
MIC: Minimal inhibitory concentration
VRE: Vancomycin resistant *E. faecalis*.
